# Effects of Nutritional Interventions on Cardiovascular Disease Health Outcomes in Aboriginal and Torres Strait Islander Australians: A Scoping Review

**DOI:** 10.3390/nu13114084

**Published:** 2021-11-15

**Authors:** Bobby Porykali, Alyse Davies, Cassandra Brooks, Hannah Melville, Margaret Allman-Farinelli, Julieann Coombes

**Affiliations:** 1 School of Public Health, Faculty of Health, University of Technology Sydney, Sydney, NSW 2007, Australia; 2 Indigenous Health Unit, Sydney Medical School, Faculty of Medicine and Health, University of Sydney, Sydney, NSW 2006, Australia; 3 Aboriginal & Torres Strait Islander Health Program, George Institute for Global Health, Newtown, NSW 2042, Australia; jcoombes@georgeinstitute.org.au; 4 Charles Perkins Centre, Nutrition and Dietetics Group, Sydney School of Nursing, Faculty of Medicine and Health, University of Sydney, Sydney, NSW 2006, Australia; alyse.davies@sydney.edu.au (A.D.); cbro2650@uni.sydney.edu.au (C.B.); hmel2900@uni.sydney.edu.au (H.M.); margaret.allman-farinelli@sydney.edu.au (M.A.-F.); 5 Faculty of Medicine, University of New South Wales, Sydney, NSW 2052, Australia

**Keywords:** Aboriginal, Torres Strait Islander, cardiovascular disease, first peoples, first nations, health promotion, nutrition

## Abstract

Nutrition interventions can support Aboriginal and Torres Strait Islander peoples to reduce their risk of cardiovascular disease (CVD). This review examines nutritional interventions aiming to improve CVD outcomes and appraises peer-reviewed interventions using an Aboriginal and Torres Strait Islander Quality Appraisal Tool. Five electronic databases and grey literature were searched, applying no time limit. Two reviewers completed the screening, data extraction and quality assessment independently. The study quality was assessed using the South Australian Health and Medical Research Institute and the Centre of Research Excellence in Aboriginal Chronic Disease Knowledge Translation and Exchange Aboriginal and Torres Strait Islander Quality Appraisal Tool (QAT). Twenty-one nutrition programs were included in this review. Twelve reported on anthropometric measurements, ten on biochemical and/or hematological measurements and sixteen on other outcome domains. Most programs reported improvements in measurable CVD risk factors, including reduced body mass index (BMI), waist circumference (WC), weight, blood pressure and improved lipid profiles. Most programs performed well at community engagement and capacity strengthening, but many lacked the inclusion of Indigenous research paradigms, governance and strengths-based approaches. This review highlights the need for contemporary nutrition programs aimed at improving cardiovascular health outcomes to include additional key cultural components.

## 1. Introduction

Aboriginal and Torres Strait Islander Australians are strongly connected to “country” (land, waterways, and seas) through family, spiritual and traditional links that contribute to their health and wellbeing. History tells us that practices, policies and legislations directed towards Aboriginal and Torres Strait Islander peoples during Australia’s early colonial years resulted in their forceful removal from lands and a disconnection from “country” that contributed to sudden changes in their dietary and lifestyle behaviours [1]. Traditionally, the nutrient composition of Aboriginal and Torres Strait Islander peoples’ diet was high in protein, fibre, polyunsaturated fats and complex unrefined carbohydrates [2]. The low energy density of traditional diets along with the energy expended in food procurement processes provided a natural constraint on energy intake, and research suggests that pre-colonisation Aboriginal and Torres Strait Islander peoples were physically fit and lean [3]. The resulting loss of traditional lands, waterways and food practices, along with food insecurity and geographic factors, has changed their diet into a heavily processed western diet [4,5,6]. A western diet has been linked to the risk of developing Cardiovascular disease (CVD) and/or other complications as the diet features high amounts of saturated fat, red meat-based protein, added sugars, salt, processed and fast-foods, as well as low levels of fibre, certain micronutrients, wholegrains, fruit and vegetables [7]. 

Cardiovascular disease is a class of disorders affecting the heart and blood vessels and includes: coronary heart disease, ischemic heart disease, cerebrovascular disease, stroke, and others [8]. Cardiovascular disease risk factors include smoking, excessive alcohol, lack of physical activity, being overweight or obese, not eating the recommended amounts of fruit and vegetables and high blood pressure, blood glucose and cholesterol [9,10]. While CVD is a leading and serious disease in Australia [11], Aboriginal and Torres Strait Islander peoples have higher rates of CVD-related hospitalization and death compared to non-Indigenous Australians [12]. Addressing modifiable risk factors, such as diet, would help to prevent 37% of the burden of disease in Aboriginal and Torres Strait Islander populations [13].

The release of the Social Justice Report in 2005 resulted in a push towards achieving equality for Aboriginal and Torres Strait Islander peoples in relation to health and life expectancy [14]. Subsequently, this led to the collaborative initiation of Close the Gap campaign and the government response, through Closing the Gap [15]. One of the main considerations articulated for achieving equality in health outcomes is that research and programs are co-developed with communities and conducted in an appropriate way suitable to the needs, culture and paradigm of Aboriginal and Torres Strait Islander peoples. Further, there is an important emphasis on taking a strengths-based and preventative health approach [16,17]. Nutrition promotion is an important part of cardiovascular-oriented health promotion and food and water security are key to allowing Aboriginal and Torres Strait Islander people to eat a healthy diet [18]. While previous health and nutrition programs have targeted Aboriginal and Torres Strait Islander peoples, none have been appropriately appraised to determine if they are culturally suitable, and few have been adequately evaluated [19,20]. Evaluations are crucial to understanding the viability, effectiveness and impact of nutrition programs as they inform nutrition policy and practice [21]. Moreover, they provide the evidence base necessary for funding and can enhance community support and awareness when released appropriately to stakeholders and the general public. 

Cultural awareness, community engagement and partnerships, combined leadership, and sustainable resources are integral components of programs that aim to improve the health of Aboriginal and Torres Strait Islander peoples [19]. However, the existing frameworks and strategies used to guide the design and evaluation of Aboriginal and Torres Strait Islander nutritional programs have lacked appropriate tools to evaluate these programs and principles [22,23]. Aboriginal and Torres Strait Islander Quality Appraisal Tools (QAT) offers a credible and rigorous assessment to ensure programs are designed and delivered in a culturally appropriate manner [24]. A scoping review was selected for its ability to map all the relevant literature, including scientific peer-reviewed publications, as well as unpublished grey literature, to fill in the gaps in knowledge on this topic [25]. This review (1) examines the effects of nutrition interventions that aim to improve CVD outcomes in Aboriginal and Torres Strait Islander peoples; and (2) assesses the cultural appropriateness of programs with scientific peer-reviewed evaluations using the Aboriginal and Torres Strait Islander QAT.

## 2. Materials and Methods

### 2.1. Protocol and Registration

The performance of this scoping review followed the Arksey & O’Malley framework [26] and the findings are reported in accordance with the Joanna Briggs Institute updated methodological guidance for scoping reviews [27]. The protocol was developed and published on the Open Science Platform https://osf.io/z38gt/, accessed on 12 April 2021.

### 2.2. Inclusion Criteria

#### 2.2.1. Participants

Aboriginal and Torres Strait Islander peoples of any age, with or without co-morbidities and residing in Australia were included. Programs were excluded if Aboriginal and Torres Strait Islander peoples did not make up more than 50% of the sample population or the data were unable to be extracted from non-indigenous Australians. 

#### 2.2.2. Concept

Programs or studies describing nutritional interventions that aim to improve CVD health outcomes were included. This included programs or studies with or without co-interventions targeting additional lifestyle diseases, as well as programs aiming to improve food security and dietary quality. Interventions that focused only on nutrient supplementation were excluded. For the published peer-reviewed studies, the interventions were required to have at least one marker from the following two outcome domains; (1) anthropometric measures (i.e., waist circumference (WC), body mass index (BMI), weight, hip measurements, waist-to-hip ratio (WHR) and fat mass); (2) biochemical and/or hematological biomarkers (i.e., total cholesterol (TC), high density lipoprotein cholesterol (HDL-C), low density lipoprotein cholesterol (LDL-C), triglycerides (TG), blood glucose level (BGL), systolic blood pressure (SBP), diastolic blood pressure (DBP), mean arterial blood pressure (MABP), fasting insulin and glycated haemoglobin (HbA1c). An additional program outcome category, “other”, was included to capture any additional information (mostly relevant for non-published programs).

#### 2.2.3. Context

This review considered programs conducted in the community at large from all the regions of Australia. This included programs delivered through outpatient facilities or clinics. Programs conducted in institutional settings (i.e., hospitals or aged care facilities) were excluded as disease or illnesses may influence dietary requirements and intake.

### 2.3. Types of Studies

To understand the extent, range and nature of all the programs aiming to improve CVD outcomes in Aboriginal and Torres Strait Islander peoples, the eligibility criteria for the included study types was broad. Primary study designs, conference proceedings, posters or abstracts, editorials, commentaries, perspectives, book chapters, newsletters, dissertations, theses, and government-produced reports and documents were all included. Systematic reviews and meta-analysis were excluded. The language of programs was restricted to English and there was no set date limit for the year of program dissemination.

### 2.4. Search Strategy

An initial limited search was conducted on MEDLINE and the Australian Indigenous HealthInfoNet to identify articles on the topic. A full search strategy was developed based on the key words contained in the titles and abstracts, as well as indexed terms used to classify the articles. A full search was conducted on Ovid (Medline and Embase), Scopus and Informit (Family and Society Collection and Indigenous Collection). Grey literature sources included the Australian Indigenous HealthInfoNet, Google Scholar and Lowitja Institute. The search strategy for MEDLINE is shown in Supplementary Table S1, conducted in March 2021. The reference lists of studies that were selected for full text screening were examined for additional programs. 

### 2.5. Selection Process

**Figure 1:**
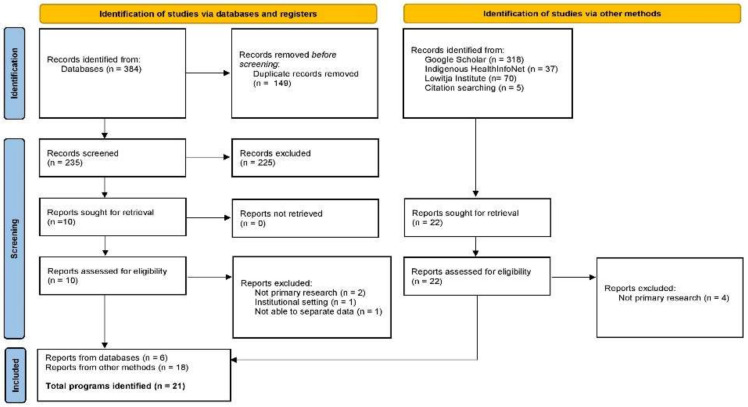
PRISMA Flow Diagram.

The identified records from the full search were imported into EndNote X9.3.3. (Clarivate Analytics, PA, USA) for screening. The titles and abstracts of the retrieved studies were screened against the inclusion criteria by two independent reviewers (C.B. and H.M.). For the studies with the potential to be included, full texts were retrieved and attached in EndNote. Two reviewers screened against the inclusion and exclusion criteria independently. The reasons for exclusion after full text screening were recorded in EndNote and any discrepancies between the reviewers that arose throughout the process was resolved through discussion or consultation with a third reviewer (A.D.). The search results are presented in a PRISMA flow diagram (Figure 1).

### 2.6. Data Charting

The data was charted using a standardized data charting form based on an existing framework for scoping reviews [26,27]. The following information was extracted for each included program: program reference, aim(s), intervention summary, timeframe (duration and time to follow up), target population, setting, outcome measures (anthropometric, biochemical and/or hematological biomarkers or other) and program status (completed, active or unclear). Using Microsoft Excel, two independent researchers (C.B. and H.M.) independently extracted data and resolved any discrepancies through discussion or consultation with a third reviewer (A.D.). In cases in which one program had multiple reports, all the reports were included if they met the inclusion criteria to ensure that the full details of the program were recorded. The authors of papers or relevant government bodies were contacted if missing or additional data needed to be requested.

### 2.7. Critical Appraisal

To assess the quality of the peer-reviewed research and its cultural appropriateness, the 2018 South Australian Health and Medical Research Institute (SAHMRI) and the Centre of Research Excellence in Aboriginal Chronic Disease Knowledge Translation and Exchange (CREATE) Aboriginal and Torres Strait Islander Quality Appraisal Tool (QAT) was implemented [24]. Two independent reviewers (C.B. and H.M.) used the Aboriginal and Torres Strait Islander Quality Appraisal Tool’s Companion Document [28] as well as the Practical Guide for Researchers from the Lowitja Institute [29] to guide their response to the 14-question QAT, with disagreements resolved through consultation with a third Aboriginal reviewer (J.C.). 

The responses to the QAT included yes, partial, unclear and no. The Yes category was assigned to studies that clearly provided written evidence in favour of the question. The Partial category was assigned to studies that provided some evidence, or brief evidence in favour of the question. The Unclear category was assigned to studies that may have actioned question items but did not explicitly report full or partial evidence. The No category was assigned to studies that did not report any evidence or reported evidence not in favour of the question item. These responses were presented in an adapted traffic light plot using the colours: green, blue, orange and red. A program was considered more culturally appropriate and of higher research quality if it received more “yes” responses. Inversely, a program was considered less culturally appropriate and of lower research quality if it received more “no” responses. A final rating of “very good” was assigned to papers that answered yes to over 75% of questions, “good” to those that answered yes to over 50% of questions, “fair” to those that answered yes to fewer than 50% of questions, and “poor” to those that answered yes to fewer than 25% of questions. To score how well each question performed, three points were assigned for “yes-green”, two points for “partially-blue”, one point for “unclear-orange” and no points for “no-red”. 

### 2.8. Synthesis of Results

The results are presented in tabular form, with an accompanying narrative summary to describe the results in relation to the scoping review’s aims. Table 1 includes programs published in grey literature, while Table 2 includes programs published in scientific peer-reviewed literature. The results were interpreted through a decolonized lens privileging Aboriginal and Torres Strait ways of knowing [30,31]. The findings of this review will be used to inform future community-led nutrition interventions aiming to improve CVD disease outcomes in Aboriginal and Torres Strait Islander peoples. 

## 3. Results

### 3.1. Search Results

The academic database search yielded a total number of 384 records using the search strategy on five electronic databases (Medline = 124, Embase = 157, Scopus = 81, Informit Indigenous Collection = 21, Informit Family and Society Collection = 1). This was reduced to 235 after 149 duplicates were removed. A total of 235 records were then screened, with 225 records excluded. Ten reports were sought for full text retrieval and assessed for eligibility. Of these ten reports, a total of six reports representing five programs were identified through databases and included in this review. 

A total of 430 records were identified via other methods, of which 22 full text reports were retrieved. Of these 22 reports, four were excluded as they were not primary research. A total of 18 reports, representing 16 programs, were identified through other methods and were included in this review. The flowchart in Figure 1 illustrates the process of the selection of sources of evidence. 

### 3.2. Program Selection and Characteristics

A total of 24 reports were included in the review, representing 21 nutrition programs. Of these 21 programs, 12 were unpublished [32,33,34,35,36,37,38,39,40,41,42,43,44] and nine programs published in the scientific peer-reviewed literature [45,46,47,48,49,50,51,52,53,54,55]. Both unpublished programs (2000 to 2020) and peer-reviewed published literature (1994 to 2014) spanned over a period of 20 years. Four programs were located in Queensland [32,39,43,46], Western Australia [34,48,49,51,54,55], Northern Territory [37,40,50,52], New South Wales [36,38,41,42] and one in Victoria [33], South Australia [45], Tasmania [47], Central Australia [53] and Australia-wide [44]. Nine programs were completed [36,37,42,43,45,46,47,51,52,53,54,55], eight were active [32,33,34,35,38,39,40,44,48,49] and the status of four was unclear [41,50,52,54,55]. The participant size ranged from 10 to over 400, but only 62% of the programs specified the total sample size. The program duration varied between one day and two years, while the time to follow-up ranged from eight weeks to eight years. One program included only female participants [36], and another included only male participants [42]. The delivery of programs was primarily in-person and group-based; however, two programs offered a telephone service [39,44] and one provided an online component [39]. The education topics predominantly included healthy eating, weight loss and management, food budgeting, food label reading, and the benefits of exercise.

### 3.3. Anthropometric Outcomes

A total of 12 programs reported on anthropometric measurements [32,42,43,45,46,47,48,49,50,51,52,53,54]. Five programs produced significant reductions in BMI [45,48,49,50,52,54], four produced significant decreases in WC [42,46,48,49,50], three reported significant decreases in weight [42,45,50] and one reported significant reductions in WHR, fat mass and body fat percentage [50]. Effect sizes were used in one program to report reductions in weight, BMI and WC [47]. At an eight-year follow-up, one program reported significant increases to women’s BMI and WC [53].

### 3.4. Biochemical and/or Haematological Outcomes

A total of 10 programs reported on biochemical and/or hematological outcomes [42,43,44,45,46,48,49,51,52,53,54,55]. Significant reduction in TC was the most common improvement in this category and was seen in four programs [44,46,52,53], while a significant reduction in the prevalence of age-adjusted hypercholesterolemia (HC) was reported in one program [54,55] and a decrease in the odds (OR 0.29) of HC at eight years’ follow-up for another [53]. Three programs produced significant reductions in HDL-C [42,46,53], while one program reported an increase, although its statistical significance is unclear [43]. One program reported significant improvements in targeted achievements in LDL-C [44]. A significant reduction in TG was reported for one program [46], while another program reported a significant increase in TG and dyslipidemia at the eight-year follow-up [53]. Three programs produced a significant decrease in SBP [42,48,49,52] and DBP [46,48,49,52]. One program reported significant reductions in BGL [42], while another program saw significant decreases in two-hour plasma glucose, fasting glucose and fasting insulin; however, these reductions were not sustained [54,55].

### 3.5. Other Outcomes

A total of 16 programs reported other outcomes [32,33,34,35,36,37,38,42,43,44,46,47,48,49,51,52,53,54,55]. Five programs reported improvements to diet quality [32,43,52,53,54,55], with the main improvement relating to increased consumption of fruits and vegetables. Three programs reported statistically significant improvements in fitness parameters and engagement in physical activity [42,44,48,49], while improvements in physical activity were reported in nine others [32,34,35,36,37,43,46,47,51,54]. Other outcomes included improvements in participants’ knowledge and skills [32,34,35,36], self-esteem [34,35], well-being [43], fatigue levels and quality of life [47].

### 3.6. Quality Appraisal

**Figure 2:**
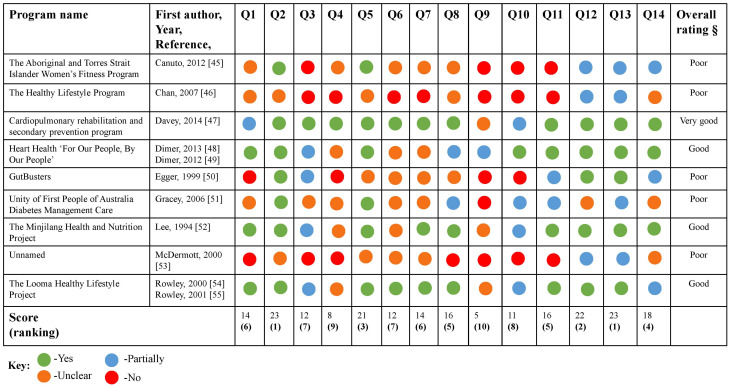
The 2018 SAHMRI CREATE Aboriginal and Torres Strait Islander Quality Appraisal Tool results of peer-reviewed programs presented as an adapted traffic light plot. Q1 Did the research respond to a need or priority determined by the community?; Q2 Was community consultation and engagement appropriately inclusive?; Q3 Did the research have Aboriginal and Torres Strait Islander research leadership?; Q4 Did the research have Aboriginal and Torres Strait Islander governance?; Q5 Were local community protocols respected and followed?; Q6 Did the researchers negotiate agreements in regards to rights of access to Aboriginal and Torres Strait Islander peoples existing intellectual and cultural property?; Q7 Did the researchers negotiate agreements to protect Aboriginal and Torres Strait Islander peoples’ ownership of intellectual and cultural property created through the research?; Q8 Did Aboriginal and Torres Strait Islander peoples and communities have control over the collection and management of research materials?; Q9 Was the research guided by an Indigenous research paradigm?; Q10 Does the research take a strengths-based approach, acknowledging and moving beyond practices that have harmed Aboriginal and Torres Strait peoples in the past?; Q11 Did the researchers plan to and translate the findings into sustainable changes in policy and/or practice?; Q12 Did the research benefit the participants and Aboriginal and Torres Strait Islander communities?; Q13 Did the research demonstrate capacity strengthening for Aboriginal and Torres Strait Islander individuals?; Q14 Did everyone involved in the research have opportunities to learn from each other? The symbol § represents the overall rating of peer-reviewed programs. A final rating of “very good” was assigned to papers that answered yes to over 75% of questions, “good” to those that answered yes to over 50% of questions, “fair” to those that answered yes to fewer than 50% of questions, and “poor” to those that answered yes to fewer than 25% of questions.

The quality appraisal results are presented in Figure 2 with green representing (yes), blue (partially), orange (unclear) and red (no) for the responses to 126 questions. Green represented 35% of all the responses, followed by orange (28%), blue (19%) and red (18%). 

The program that performed best in terms of overall cultural appropriateness was by Davey et al. 2014 [47]. Three programs were rated as good [48,49,52,54,55] and the remaining five programs were rated as poor [45,46,50,51,53]. No trend was apparent regarding the date of programs and the ranking of cultural appropriateness, despite the most recent program receiving the highest ranking [47].

The three questions that received the highest score across all the programs were: Question 2, “Was the community consultation and engagement appropriately inclusive?”; Question 12, “Did the research benefit the participants and Aboriginal and Torres Strait Islander communities?”; and Question 13, “Did the research demonstrate capacity strengthening for Aboriginal and Torres Strait Islander individuals?”. By contrast, the three lowest-performing questions were; Question 4, “Did the research have Aboriginal and Torres Strait Islander governance?”; Question 9, “Was the research guided by an Indigenous research paradigm?" and Question 10, “Does the research take a strengths-based approach, acknowledging and moving beyond practices that have harmed Aboriginal and Torres Strait Islander peoples in the past?”.

## 4. Discussion

This scoping review is the first to map the evidence relating to the effectiveness of nutrition programs aiming to improve CVD outcomes in Aboriginal and Torres Strait Islander peoples and to appraise peer-reviewed interventions using a culturally appropriate Aboriginal and Torres Strait Islander QAT. Among the 21 programs identified, 12 programs were from unpublished research and nine from published research. Most programs reported improvements in measurable risk factors for CVD, including reduced BMI, WC, weight, blood pressure and improved lipid profiles. Other improvements included knowledge, skills, well-being and quality of life. It appears that a multifaceted education approach was consistently implemented. The predominant topics included healthy eating, weight loss and management, food budgeting, food label reading, and the benefits of exercise. Incorporating physical activity components was a common feature of many of the programs, which likely contributed to improved metabolic control. From the nine peer-reviewed publications that were critically appraised and evaluated using the QAT, four programs that were deemed more culturally appropriate were likely to be associated with program sustainability [47,48,49,52,54,55] and may still be active within communities [48,49,52,54,55]. 

Program evaluations help us to demonstrate impact and improve program design and implementation, as well as to drive support for funding. This scoping review found that not all programs had been evaluated and that of those that were evaluated, few were peer-reviewed. Peer-reviewed programs offered greater detail on anthropometric and biochemical and/or haematological measurements than non-peer-reviewed programs. Despite the limited number of peer-reviewed programs, the outcomes generally supported previous findings in that targeted cardiovascular programs incorporating both nutrition and physical activity are beneficial in reducing CVD risk and improving clinical outcomes [56,57]. Other qualitative and behavioural outcomes lacked consistency, were reported less frequently and were often based on anecdotal remarks. In the past, cultural components were not included as evaluable outcomes within nutrition programs for CVD [58]. A mixed method approach is often suitable for Aboriginal and Torres Strait Islander research, as qualitative feedback can provide valuable insight into the cultural relevance and acceptability of program components [59], factors of which can influence outcome measures. The most recent program in 2014 by Davey and colleagues rated best for overall cultural appropriateness [47] and was the only peer-reviewed program that reported improved quality of life and fatigue levels. 

Social determinants are estimated to contribute to over 30% of the health gap between Aboriginal and Torres Strait Islander peoples and non-Indigenous Australians [17]. Those with higher levels of education, income and food security have better health outcomes [60]. Interventions that provided multiple education components that address social determinants were identified to be effective in reducing measures across more than one anthropometric outcome. These educational components increased knowledge and awareness in the areas of healthy eating, weight loss and management, food budgeting, food label reading and the benefits of exercise. Consistent with a holistic health approach, additional education messaging was delivered on health literacy, wellbeing and stress management. 

Another key program characteristic that appeared to correspond with improved outcome measures was a longer timeframe designated for interventions. A program that reduced measures in all the anthropometric outcomes of BMI, WC, weight, WHR and fat reduction was delivered over 12 months [50]. Similarly, the two interventions that performed well at reducing measures within multiple biochemical/haematological outcomes were delivered across communities over a longer time-frame of 2 years [46] and 8 years [53]. Emerging evidence suggests that long-term changes in diet quality correlate with a lower risk of CVD and mortality [61]. Interestingly, the three programs that performed well across clinical measures scored ‘poor’ in the quality appraisal [46,50,53]. 

Scholarship in the areas of Indigenous epistemology, ontology and axiology has led to an understanding of Aboriginal and Torres Strait Islander ways of knowing as fluid, meaning that it can evolve and are influenced by situational contexts, such as the political and social environment [30,31]. The timeline towards healing and reconciliation, as seen through the social justice report and the Close the Gap and Closing the Gap campaigns have established a discourse amongst academics and researchers to promote Indigenous paradigms in research [14,15,30]. This privileging of Aboriginal and Torres Strait Islander knowledge has led to the development of quality appraisal tools to assess programs not only through a scientific lens but also through an Aboriginal and Torres Strait Islander cultural lens [24]. 

This review retrospectively assessed the cultural appropriateness of programs and highlighted that of the programs evaluated and peer-reviewed, less than half of the programs answered ‘yes’ to 50% or more of the quality appraisal questions. As evidenced, most programs performed well at community engagement and capacity strengthening but they lacked the inclusion of Indigenous research paradigms, governance and taking a strengths-based approach. The lack of inclusion of cultural components within the program should be interpreted with consideration that they were reported between 1994 and 2014 and the Aboriginal and Torres Strait Islander QAT used herein was developed in 2018 and published in 2020 [24]. It is possible that the ‘poor’ QAT score assigned to programs is reflective of the scholarship and understanding in Aboriginal and Torres Strait Islander research at that time. Importantly, this indicates a gap in research and highlights a need for contemporary nutrition programs aimed at improving cardiovascular health outcomes to include additional key cultural components. 

An extensive and wide search was a strength of this review, scoping all the available published and unpublished literature. Further, to the author’s knowledge, this review was the first to appropriately apply a quality appraisal tool from an Indigenous perspective to ascertain the cultural appropriateness of nutrition programs targeting CVD. A limitation to the review was that only a small number of programs was evaluated, which resulted in limited application of the QAT. Furthermore, this scoping review retrospectively assessed the cultural appropriateness of programs as most peer-reviewed studies retrieved were published between 1994 to 2014, before the development of the QAT in 2018. It is important that the value and relevance of the previous research and programs presented in this review are acknowledged. The findings are not a reflection of overall poor cultural appropriateness, but rather a reflection of the advancement of knowledge in the areas of Indigenous epistemology, ontology, axiology and research methodologies.

## 5. Conclusions

Future nutrition programs aiming to improve cardiovascular health outcomes should be multifaceted, incorporating both nutrition and physical activity. Most programs identified in this review undertook community consultation, engagement and capacity strengthening, but improvements in key areas of Indigenous paradigm, governance and strengths-based approaches are required. Future research should focus on sustainability within communities through effective co-design with Aboriginal and Torres Strait Islander peoples and communities. 

## Figures and Tables

**Table 1:** Program characteristics for unpublished literature (*n* = 12).

Program Name, Reference, Year	Aims	Intervention Summary	Timeframe (Duration/Time to Follow-up)	Target Population	Setting	Anthropometric Measurements	Biochemical and/or Haematological Biomarkers	Other Outcomes	Status
Living Strong (formerly known as the Healthy Weight Program) [32] 2005	To help Aboriginal and Torres Strait Islander peoples maintain a healthy weight and prevent lifestyle diseases	Total participants (*n* = 432) Follow up (*n* = 34) QLD government-initiated weight management and healthy lifestyle program designed to teach lifestyle skills relating to nutrition, PA and self-esteem Delivery: In-person and group-based Education: Healthy weight and weight loss; behaviour change; low fat cooking; budgeting for healthy meals; shopping tour; self-esteem; benefits of exercise; diabetes awareness	3 months/ 0, 8, 12 wks (baseline, mid, post program)	Aboriginal and Torres Strait Islander adults	QLD (Program held across 8 communities; Chermside, Rockhampton, Mount Morgan, Blackwater, Gehgre, Woorabinda, Toowoomba, Wynnum)	↓ Weight (in >50% of participants) ↓ WC (in >50% of participants)	N/A	↑ Proportion of participants eating at least two daily servings of fruit ↑ Proportion of participants eating five daily servings of vegetables ↑ Reading NIP ↑ Water ↑ Using reduced fat foods ↑ PA (planned and incidental)	Active
Life! Aboriginal Road to Good Health [33] 2016	To reduce the risk of developing T2DM and CVD in Aboriginal and Torres Strait Islander peoples	Total participants (unspecified) Aboriginal and Torres Strait Islander healthy lifestyle program, supported by the Victorian government, within the “Life! Program”. The program is run by Aboriginal health workers and Aboriginal health services and included dietitians, diabetes educators and personal trainers. Culturally appropriate supporting resources were used to assist in topic delivery including facilitator manuals, participant workbook material including recipes and healthy eating and exercise books/posters Delivery: six sessions delivered in-person as a group course (telephone health coaching service or the group course via zoom available during the coronavirus pandemic) Education: healthy eating; maintaining a healthy weight; food label reading; purchasing healthy/cost effective foods; benefits of exercise; diabetes prevention; cessation of smoking	6 wks/nil	Aboriginal and Torres Strait Islander individuals, families and community groups. Eligibility >18 and diagnosed with one or more of the following: (heart disease or stroke, gestational diabetes, high cholesterol, BP, BGL, polycystic ovarian syndrome)	VIC (Melbourne)	N/A	N/A	↑ Engagement Feedback (the flexible, fun, family-friendly approach in addition to a passionate facilitator were key factors to its success)	Active
Pilbara Aboriginal Heart Health Program [34,35] 2017/2018	To provide comprehensive, coordinated, integrated and culturally appropriate health education services to the Aboriginal and Torres Strait Islander peoples	Total participants (unspecified) Program developed in collaboration with the Heart Foundation and Karratha Central Healthcare. Incorporates culturally relevant strategies including yarning to support positive behaviour change. The program is committed to the following principles: community control, holistic approach, cultural safety, equality, reciprocity and inclusion, best practice, building community capacity, accountability, sustainability. Delivery: group-based, in-person activities and meetings tailored to each community. Education: healthy eating; heart health; accessing health services; benefits of exercise	Unspecified	Aboriginal and Torres Strait Islander peoples	WA (3 communities in the Pilbara region: Karratha, Onslow and Roebourne)	N/A	N/A	↑ Self-esteem ↑ Knowledge (healthy eating) ↑ PA ↑ CVD health knowledge	Active
Healthy Eating Activities and Lifestyles for Indigenous Groups (HEALInG) [36] 2007	To provide realistic and practical information on healthy eating and lifestyle activities to support weight loss	Total participants (*n* = 11) Goonellabah (*n* = 3) GurgunBulahnggelah (*n* = 6 to 8) Program adapted from QLD Health’s Healthy Weight Program. Delivery: in-person and group-based. One hr exercise class, followed by an education session on healthy eating and/or lifestyle topics Education: dietary guidelines; food groups; serving sizes; reducing fat, salt and sugar intake; food budgeting and label reading; benefits of exercise. Information was also provided on CVD, diabetes and stroke prevention	10 wks/ 0,10 wks	Aboriginal and Torres Strait Islander women	NSW (Northern Rivers region including Goonellabah and GurgunBulahnggelah)	N/A	N/A	↑ Knowledge and skills relating to: cooking healthy and budget conscious meals; achieving daily PA targets; food label reading; fats, sugar, and salt content within foods; chronic diseases; and the positive effects of diet and exercise	Completed
Cooking Healthy and Physical Activity (CHAPA) Project [37] 2009	To encourage active self-management for people with T2DM and/or heart disease	Total participants (unspecified) Group 1 (*n* = 15) Program is one of four programs from Healthy Active Australia “Community and School Grants Program”. Multidisciplinary team including dietitians, exercise physiologists and psychologists. Delivery: in-person and group-based Education: goal setting; healthy eating and cooking; benefits PA	10 wks/nil (four groups, over 12 months)	People with heart disease and/or T2DM	NT (greater Darwin region)	N/A	N/A	Anecdotal remarks were made on improvements in health, fitness, weight, BP and functional strength	Completed
Koori Cook Off Program [38] 2017	To improve heart health outcomes through nutrition education and increase confidence, knowledge and skills of healthy cooking and eating	Total participants (unspecified) A cooking challenge program developed by and for Aboriginal communities in collaboration with the Heart Foundation. Using healthy foods, groups cook healthy meals for a panel of judges (local Elders) Delivery: in-person and group-based, four teams, each with four people Education: culinary skills; creating healthy meals using basic ingredients; portion sizes; practical ways to increase FV consumption; healthier oils; reducing salt; choosing mainly water	nil/nil	Aboriginal and Torres Strait Islander peoples	NSW (Communities throughout the Illawarra and Shoalhaven regions)	N/A	N/A	Anecdotal feedback suggested that the program was popular with the community	Active
My Health for Life (MH4L) [39] 2016	To decrease participants’ risk of developing conditions such as T2DM, heart disease, stroke, high cholesterol and high BP	Total participants (unspecified) Initiated by QLD government, now run by a NGO partnership including Diabetes QLD, National Heart Foundation, Stroke Foundation and others. Participants must complete a health check to participate. A culturally appropriate tailored version of the program was available for Aboriginal and Torres Strait Islander peoples Delivery: six-session program delivered either remotely via telephone or group-based and in-person. This followed by a six-month online maintenance program Education: healthy eating; benefits of exercise; weight management; consuming safe levels of alcohol; smoking cessation	6 months/nil	Aboriginal and Torres Strait Islander peoples at risk of developing T2DM, heart disease, stroke, high cholesterol and high BP	QLD (statewide)	N/A	N/A	N/A	Active
Gudbinji Chronic Disease Program [40] N/A	To improve heart health and heart health risk factors	Total participants (unspecified) A one-day heart health focused program that is part of a larger 10 wk chronic disease (including CVD) program Delivery: In-person Education: Healthy eating and cooking with dietitians; benefits of exercise	1 day/nil	Aboriginal and Torres Strait Islander peoples	NT (Katherine, Wurli-Wurlinjang Aboriginal Health Service)	N/A	N/A	N/A	Active
Aboriginal and Torres Strait Islander Heart Care Project “Urimbirra Geen” [41] 2000	To improve heart disease risk factors and other factors affecting the health of Aboriginal and Torres Strait Islanders	Total participants (unspecified) This multi-faceted project is a partnership between government and NGOs addressing heart health issues within the local Aboriginal and Torres Strait Islander community Delivery: Through involvement with a variety of different community initiatives including: youth athletics carnival; “Yandarra”—a healthy lifestyle partnership project; Wagga Wagga Elders Physical Activity Group; culturally appropriate educational materials distributed by the Greater Murray Area Health Service Education: depend on the community initiative involved but was centred around healthy eating; packing healthy lunches and snacks; PA; tobacco and alcohol	N/A	Aboriginal and Torres Strait Islander peoples	NSW (Wagga Wagga)	N/A	N/A	N/A	Unclear
Strong Men [42] 2020	To decrease CVD risk factors in Aboriginal men aged 35–80 yrs and understand the experience of Aboriginal men who participated in the exercise and health education program	Total participants (*n* = 10) A short program focused on cardiovascular exercise and health education. Program was developed with local Aboriginal community input Delivery: in-person and group-based Education: nutrition and healthy eating; social and emotional well-being; PA	10 wks/10 wks	Aboriginal and Torres Strait Islander men (35–80 yrs)	NSW (Albury Wodonga)	↓ WC **^‡^ ∆ 123.5 cm (24.9) to 114.5 cm (17.5) ↓ Weight *^‡^ ∆ 106.7 kg (37.7) to 104.6 kg (33.0) ↓ BMI	↓ TC ↓ HDL-C *^‡^ ∆ 1.25 mmol/L (0.45) to 1.10 mmol/L (0.27) ↓ LDL-C ↓ TG No change HbA1c ↓ BGL **^‡^ ∆ 5.58 mmol/L (3.45) to 5.25 mmol/L (1.67) ↓ SBP **^‡^ ∆ 140 mmHg (19.0) to 132 mmHg (19.2) ↑DBP ^‡^	↑ 6 min walk test **^‡^ ∆ 360 m (40) to 400 m (75) ↑ Squats/1 min **^‡^ ∆ 29.0 (7.7) to 45.5 (12.2) ↑ Incline push ups/1 min **^‡^ ∆ 28.5 (5.5) to 39.5 (8.0) ↑ Shoulder press/1 min **^‡^ ∆ 44.5 (22.0) to 68.5 (30.7) ↑ Step ups/1 min **^‡^ ∆ 24.0 (9.5) to 36.5 (13.0)	Completed
Walkabout Together [43] 2006	To address high community levels of chronic disease (including hypertension and diabetes) via a nutrition and PA lifestyle program	Total participants (*n* = 150) Follow up (*n* = 126) Townsville Aboriginal and Islanders Health Services developed this lifestyle modification program to reduce impact of chronic disease in the community Delivery: in-person and group-based weekly support sessions. Patients had access to GPs, dietitians and health workers for regular check-ups. Regular recreational walks were encouraged Education: unspecified	1 yr/1 yr	Aboriginal and Torres Strait Islander peoples (overweight; BMI > 25 kg/m^2^)	QLD (Townsville Aboriginal and Islanders Health Service)	↓ Weight ↓ WC	↓ BGL ↓ TC ↑ HDL-C ↓ TG ↓ DBP No change to SBP and HbA1c	Food/Nutrient intake: ↑ Participants consuming the recommended food group serves from AGHE PA: ↑ Participants performing moderate and vigorous PA >2 two days/wk ↑ Steps Correlation* between daily steps and moderate and vigorous PA ↓ Sedentary behaviours Other: ↑ Wellbeing *	Completed
Coaching Patients on Achieving Cardiovascular Health (COACH) Program [44] 2017	To reduce CVD risk	Total participants (*n* = 492) (included both Indigenous and non-Indigenous) distributed through the QLD Health Contact Centre, this program is the first standardized coaching program targeting CVD risk factors via telephone and mail outs Delivery: five coaching sessions by trained health professionals over 6 months, in form of telehealth (phone calls) and mail outs Education: lifestyle modification; goal setting; disease management	6 months/unspecified	Patients with CHD and/or T2DM	Australia wide	N/A	↓ TC *** ↓ LDL-C *	↑ PA ***	Active

AGHE = Australian guide to healthy eating, BGL = blood glucose level, BMI = body mass index, BP = blood pressure, CHD = coronary heart disease, cm = centimetres, CVD = cardiovascular disease, DBP = diastolic blood pressure, FV = fruit and vegetables, GP = general practitioner, HbA1c = glycated haemoglobin, HDL-C = high density lipoprotein cholesterol, hr = hour(s), IQR = Interquartile range, kg = kilogram, LDL-C = low density lipoprotein cholesterol, m = meters, min = minutes, MmHg = millimeters of mercury, mmol/L = millimoles per litre, N/A = not applicable, NGO = non-government organisation, NIP = Nutrition Information Panel, NSW = New South Wales, NT = Northern Territory, PA = physical activity, QLD = Queensland, SBP = systolic blood pressure, SD = standard deviation, TC = total cholesterol, TG = triglyceride, T2DM = type 2 diabetes mellitus, VIC = Victoria, WA = Western Australia, WC = waist circumference, wk = week(s), year = yr(s), * ≤0.05, ** ≤0.01, *** ≤0.001, ‡ median (IQR), ∆ change, ↓ decrease, ↑ increase.

**Table 2:** Program characteristics for published literature (*n* = 9).

Program Name, Reference, Year	Aims	Intervention Summary	Timeframe (Duration/Time to Follow-up)	Target Population	Setting	Anthropometric Measurements	Biochemical and/or Haematological Biomarkers	Other Outcomes	Status
The Aboriginal and Torres Strait Islander Women’s Fitness Program [45] 2012	To evaluate the impact of the program on WC, weight and biomarkers from baseline (T1) to immediately post program (T2) and to assess if outcomes were maintained at 3 month follow-up (T3)	Randomised Controlled Trial, Total participants (*n* = 100) Significant lost to follow up and missing data. An exercise and nutrition program. The cohort was split between an active group and a waitlisted group (control) Delivery: in-person and group-based. Two one-hr cardiovascular and resistance training classes per wk and four nutrition education workshops Education: food label reading; recipe modification; cooking demonstration	12 wks/ 12 wks (T2) and 3 months (T3)	Aboriginal and/or Torres Strait Islander women aged 18 to 64 yrs Participants must have had a WC >80 cm Pregnant or breastfeeding (excluded)	SA (Adelaide)	Association between active group and control, adjusting for all potential confounders and T2DM (T2) ↓ Weight *^β^ ∆1.65 kg ↓ BMI *^β^ ∆ 0.66 kg/m^2^ ↓ Waist and hip measurements (T3) ↓ Weight *^β^ ∆ 2.50 kg ↓ BMI **^β^ ∆ 1.03 kg/m^2^ ↓ Waist and hip measurements	Association between active group and control, adjusting for all potential confounders and T2DM (T2) ↓ SBP ↓ DBP ↓ HbA1c ↓Glucose ↓Insulin TC (no change) ↑TG ↑ LDL-C ↓ HDL-C ↓ CRP (T3) ↓ SBP ↓ DBP ↓ HbA1c ↓Glucose ↑Insulin ↓ TC ↓ TG ↑LDL-C ↓ HDL-C ↓ CRP	N/A	Completed
The Healthy Lifestyle Programme (HELP) [46] 2007	To determine the effectiveness of lifestyle intervention on improving diabetes and cardiovascular risk factors	Cohort study, total participants (*n* = 101) Follow up (*n* = 80) A community-based, culturally appropriate, lifestyle intervention to improve cardiovascular risk factors. Included: Self-monitoring of BGLs and PA Delivery: unspecified Education: unspecified	2 yrs/6 months (during intervention)	Aboriginal and Torres Strait Islanders, overweight, >20 yrs, ±T2DM	QLD (North Stradbroke Island and Redland Bay)	↓ WC **^†^ ∆3.1 cm ↓ BMI ↓WHR	↓ DBP **^†^ ∆ 4.6 mmHg ↓SBP ↓ MABP *^†^ ∆ 4.2 mmHg ↓ TC **^†^ ∆ 0.26 mmol/L ↓ TG *^†^ ∆ 0.18 mmol/L ↓ HDL-C ***^†^ ∆ 0.09 mmol/L ↓LDL-C ↑ HbA1c *** † ∆ 0.31% ↑ Fasting BGL	↑ Steps	Completed
Cardiopulmonary rehabilitation and secondary prevention program [47] 2014	To improve health outcomes of Aboriginal and Torres Strait Islanders with diagnosed CVD and/or associated risk factors	Cohort study, total participants (*n* = 92) Follow up (*n* = 72) Cardio-pulmonary programs (*n* = 13) developed and delivered under an Aboriginal community-controlled health service. It had two components: education and exercise Delivery: in-person and group-based. Two one-hr exercises and one one-hr education session per wk Education: CVD; benefits of exercise; shopping; cooking and eating healthy food; medication usage; risks of smoking; stress reduction techniques	8 wks/ 8 wks	Aboriginal and/or Torres Strait Islanders with a diagnosis of COPD, IHD or CHF, and at least two cardiovascular risk factors (smoking, obesity, hypertension, diabetes, dyslipidemia)	TAS (Launceston and Hobart)	Participants with risk factors ↓ Weight ∆ 0.8 kg (ES = 0.04) ↓ BMI ∆ 0.3 kg/m^2^ (ES = 0.03) ↓ WC ∆ 3.0 cm (ES = 0.17)	N/A	Participants with risk factors ↑ Six-min walk distance † ∆ 43.6 m (ES = 0.10) ↓ Dyspnoea ↓ Fatigue ↑ Quality of life	Completed
Heart Health—For Our People, by Our People [48,49] 2013/2012	To evaluate the uptake and effects on lifestyle, and cardiovascular risk factors, of cardiac rehabilitation at an AMS	Cross sectional study, total participants (*n* = 120), 18 months since program commencement A culturally appropriate cardiac rehabilitation program focused on improving cardiovascular health through nutrition, PA and lifestyle Delivery: in-person and group-based. Weekly education sessions utilising yarning and PA Education: healthy eating; heart health risk factor modification; diabetes; management of medications; healthy tucker; healthy weight; oral health; stress and emotion management; PA	Unspecified/8 wk snapshot data of 28 participants (20 female)	Aboriginal and Torres Strait Islander peoples with or at risk of chronic disease	WA (Derbarl Yerrigan Health Service, Perth)	↓ BMI *^†^ ∆ 34.0 kg/m^2^ (5.1) to 33.3 kg/m^2^ (5.2) ↓ WC **^†^ ∆ 113 cm (14) to 109 cm (13) ↓ Weight	↓ SBP *^†^ ∆ 135 mmHg (20) to 120 mmHg (16) ↓ DBP *^†^ ∆ 78 mmHg (12) to 72 mmHg (5)	↑ 6 min walk distance ** ∆ 296 m (115) to 345 m (135)	Active
Gut Busters [50] 1999	To promote long-term lifestyle changes and enable community ownership and continuation	Cohort study, total participants (*n* = 57) Follow-up (*n* = 47) An NSW Health program developed in 1991, adapted for Aboriginal and Torres Strait Islander men. A “waist loss” program that made sustainable lifestyle changes across the community through the recruitment of Indigenous male leaders Delivery: in-person and group-based Education: reducing fat intake; increasing dietary fibre; increasing daily movement; changing ‘obesogenic’ habits	12 months/2, 6 and 12 months (during intervention)	Aboriginal and/or Torres Strait Islander men	NT (4 island groups in the Torres Strait region of Northern Australia)	↓ Weight ***^†^ ∆ 107 kg (18.2) to 103 kg (18.1) ↓ WC ***^†^ ∆ 118 cm (13.6) to 114 cm (13.9) ↓ BMI ***^†^ ∆ 34.7 kg/m^2^ (5.4) to 33.6 kg/m^2^ (5.4) ↓ WHR ***^†^ ∆ 1.05 (0.05) to 0.98 (0.05) ↓ Fat mass ***^†^ ∆ 36.7 kg (12.2) to 32.8 kg (12.5) ↓ Body Fat (%) ∆ 34 (4.9) to 32 (5.6) ***^†^	N/A	N/A	Unclear (may still be active in the community)
Unity of First People of Australia Diabetes Management Care Program (UFPA) [51] 2006	To prevent chronic diseases, including CVD, T2DM and obesity	Cohort Study, total participants (unspecified) Population of sum for the four communities (*n* = 1350) An Aboriginal-run screening and intervention program designed to increase awareness of chronic disease; promote healthier living; increase screening; advocate for early treatment; increase medication compliance; reduce further health complications Delivery: community-based and in-person. Group and individual level intervention. Education: diabetes education; PA; diet and nutrition; lifestyle; self-management; weight reduction; health education	Unspecified, “many months to 3 yrs in the different communities”/Unspecified	Aboriginal and Torres Strait Islanders of all ages	WA (4 communities Gibson Desert in Pilbara, Fitzroy Valley West Kimberley, and two communities inEast Kimberley)	Findings reported for one Kimberly community (better findings reported for diabetic rather than non-diabetic persons): ↓ Weight ↓ BMI ↓ WC	Findings reported for one Kimberly community: ↓ HbA1c ↓ TC ↓ LDL-C ↑ HDL-C	↑ PA	Completed
The Minjilang Health and Nutrition Project [52] 1994	To measure nutritional status of adults at Minjilang and describe community dietary intake, and use data for planning, implementing, and monitoring and evaluation of intervention	Cohort study, total participants Minjilang (*n* = 154) Control community (*n* = 310) (during intervention period) Program included health screening (voluntary), intervention and evaluation against a comparison community Delivery: community-delivered via Minjilang Clinic Education: encouraged FV intake and lean meats similar to traditional bush foods; discourage T/A and sugary foods; exercise was encouraged	12 months/3, 6, 9, 12 (during intervention)	Aboriginal and Torres Strait Islanders adults	NT (Minjilang, Croker Island)	↓ BMI ***	↓ DBP *** ↓ SBP *** ↓ TC ***^†^ ∆12.3% ↓ Fasting TG (nondiabetics only) ↑ RBC ↑ Serum folate ↑ Serum B_6_ ↑ Plasma B-carotene ↑ Plasma ascorbic acid	↑ FV ↓ Sugar ↑ low sugar drinks ↓ T/A food ↑ Wholemeal bread ↓ %E from total and saturated fat MUFA and PUFA oils replaced other oils ↓ %E from sugars ↑ Dietary density of ascorbic acid, b-carotene, thiamine, folate, calcium ↑ Fibre	Unclear (may still be active in the community)
Unnamed [53] 2000	To raise community awareness of diabetes and CVD	Total participants (1987 *n* = 348, 1991 *n* = 331, 1995 *n* = 305) Community-based nutrition awareness healthy lifestyle program Delivery: unspecified Education: diabetes awareness; healthy food-buying	2 yrs/3,8 yrs	Aboriginal community members > 15 yrs	Central Australia	At 8 yr follow up ↑ Obesity (OR: 1.84) Women (change over time) ↑ BMI ***^†^ ↑ WC ***^†^	8 yr follow-up ↑ Dislipidemia (OR: 4.54) ↓ HC (OR: 0.29) Men (change over time) ↓ HDL-C ***^†^ ↓ TC *^†^ ↑TG **^†^ Women (change over time) ↓ HDL-C ***^†^ ↓ TC ***^†^ ↑ TG ***^†^	Food/Nutrient intake: ↓ % E from fat and saturated fat ↓ % E from sugar ↑ Complex CHO intake Store turnover method: ↓ FV ↓ Sugar ↑ Flour and bread	Completed
The Looma Healthy Lifestyle Project [54,55] 2000/2001	To reduce CHD through dietary modification	Cross-sectional study, total participants (*n* = 49) (32 intervention, 17 control) Cross-sectional community samples (baseline *n* = 200, two-yrs *n* = 185, four yrs *n* = 132) Delivery: in-person and group-based. Education: healthy cooking techniques; sources of refined CHO; importance of FV; store tours; benefits of exercise	2 yrs/ 2, 6, 12, 18, 24 months (during intervention)	Aboriginal and Torres Strait Islanders at high risk of developing diabetes and CHD	WA (Looma Aboriginal community, in the remote Kimberley region)	↓ BMI *** at 6 months	↓ Fasting plasma glucose *^†^ but returned to baseline at 12 months ∆ 0.9 mmol/L ↓ 2-hr plasma glucose **^†^ but returned to baseline at 12 months ∆ 1.6 mmol/L ↓ Fasting insulin **^†^ at 18 months Cross-sectional survey data between 1993–1997, on wider community: ↓ TC (15–34 yrs) ↓ HC *** (age-adjusted prevalence at baseline (31%), 2 (21%) and 4-yrs (15%) ↑ Plasma α-tocopherol ↑ Plasma lutein and zeaxanthin ↑ cryptoxanthin ↑ β-carotene	↓ Total and Saturated fat ↑ FV ↑PA	Unclear (may still be active in the community)

AMS = Aboriginal Medical Service, BGL = blood glucose level, BMI = body mass index, CHD = coronary heart disease, CHO = carbohydrate, cm = centimetres, COPD = chronic obstructive pulmonary disease, CRP = C-reactive protein, CVD = cardiovascular disease, DBP = diastolic blood pressure, %E = percent energy; ES = effect size, FV = fruit and vegetables, HbA1c = glycated haemoglobin, HC = hypercholesterolemia, HDL-C = high density lipoprotein cholesterol, hr = hour(s), IHD = ischaemic heart disease, IQR = Interquartile range, kg = kilogram, LDL-C = low density lipoprotein cholesterol, m = meters, MABP = mean arterial blood pressure, min = minutes, MmHg = millimeters of mercury, mmol/L = millimoles per litre, MUFA = monounsaturated fatty acids, N/A = not applicable, NSW = New South Wales, NT = Northern Territory, OR = odds ratio, PA = physical activity, PUFA = polyunsaturated fatty acids, QLD = Queensland, RBC = red blood count, SA = South Australia, SBP = systolic blood pressure, SD = standard deviation, T/A = Takeaway, TAS = Tasmania, TC = total cholesterol, TG = triglyceride, T2DM = type 2 diabetes mellitus, WA = Western Australia, WC = waist circumference, WHR = waist to hip ratio, wk = week(s), year = yr(s), * ≤0.05, ** ≤0.01, *** ≤0.001, † mean (SD), β beta-coefficient, ∆ change, ↓ decrease, ↑ increase.

## Supplementary Materials

The following are available online at https://www.mdpi.com/article/10.3390/nu13114084/s1. Table S1: Medline Search Strategy.
