# Supplementary material for: Effects of Nutritional Interventions on Cardiovascular Disease Health Outcomes in Aboriginal and Torres Strait Islander Australians: A Scoping Review
Source: Nutrients. 2021 Nov 15;13(11):4084. doi: 10.3390/nu13114084 (PMC8620344; doi:10.3390/nu13114084)
Supplement: Supplementary file 1 [file nutrients-13-04084-s001.zip › nutrients-1436010-supplementary.pdf]

Supplementary Table S1. Medline Search Strategy.

Search	Query	Records retrieved
#1	Nutrition*.tw. OR Nutrient*.tw. OR Macronutrient*.tw. OR Nutrition Assessment/ OR Nutrition Policy/ OR Nutrition Surveys/ OR Diet Surveys/ OR Diet Records/ OR Energy Intake/ OR Exp Diet/ OR diet*.tw. OR Eating/ OR eat*.tw. OR exp Food/ OR Food*.tw. OR Food Preferences/ OR exp Health Promotion/ OR Health Promotion.tw.	2420424
#2	Cardiovascular Disease/ OR Cardiovascular.tw. OR CVD.tw. OR Cardiac.tw. OR Cardiolog*.tw. OR exp Heart Diseases/ OR Heart.tw. OR Coronary*.tw. OR CHD.tw. OR Ischaemic.tw. OR IHD.tw. OR Myocardial.tw. OR exp Vascular Diseases/ OR Vascular.tw. OR Atherosclerosis.tw. OR Arter*.tw. OR Stroke.tw. OR Haemmorrhage.tw. OR exp Cardiovascular Physiological Phenomena/	4155409
#3	Aborigin*.tw OR Torres Strait Island*.tw OR Indigen*.tw. OR First People*.tw. OR First Nation*.tw.	49173
#4	Austral*.tw. OR Northern Territor*.tw. OR Victoria*.tw. Queensland* OR New South Wales.tw. OR Tasmania.tw.	171563
#5	1 AND 2 AND 3 AND 4	124
